# Associations between combinations of job demands and job control among 6,16,818 people aged 55–64 in paid work with their labour market status 11 years later: a prospective cohort study

**DOI:** 10.1007/s00420-021-01717-8

**Published:** 2021-06-07

**Authors:** Kristin Farrants, J. Head, E. Framke, R. Rugulies, K. Alexanderson

**Affiliations:** 1 Division of Insurance Medicine, Department of Clinical Neuroscience, Karolinska Institutet, SE-171 77 Stockholm, Sweden; 2 Department of Epidemiology and Public Health, University College of London, London, UK; 3 National Research Centre for the Working Environment, Copenhagen, Denmark; 4 Department of Psychology, University of Copenhagen, Copenhagen, Denmark; 5 Department of Public Health, University of Copenhagen, Copenhagen, Denmark

**Keywords:** Extending working lives, Psychosocial working environment, Job demands, Job control

## Abstract

**Background:**

Given current discussions about extending working lives, more knowledge is needed on working conditions associated with labour market status in older age.

**Objective:**

To explore associations between combinations of job demands and job control among workers aged 55–64 years and their labour market status 11 years later.

**Methods:**

A population-based prospective cohort study using nationwide register data. The 616,818 individuals in Sweden aged 55–64 who in 2001 were in paid work were categorised using a job exposure matrix based on tertiles (reference = medium control/medium demands). Participants were followed up in 2012 regarding their main labour market status (paid work, old-age pension, no income/social assistance, sickness absence/disability pension, emigrated, dead; reference = old-age pension) using multinomial logistic regression for odds ratios (OR) and 95% confidence intervals (CI). The fully adjusted analyses included adjustment for sociodemographic factors and unemployment or sickness absence/disability pension for more than half the year in 2001.

**Results:**

Those in occupations with low job control at baseline were less likely to be in paid work at follow-up (OR low demands/low control 0.74, CI 0.71–0.78; high demands/low control 0.81, CI 0.75–0.87). Those in occupations with baseline high demands were less likely to have no income/social assistance at follow-up (OR high demands/low control 0.71, CI 0.52–0.96; high demands/high control 0.59, CI 0.47–0.75).

**Conclusion:**

Job demands and control when aged 55–64 were associated with labour market status 11 years later: high control was associated with greater chance of being in paid work, and high demands were associated with lower risk of no income/social assistance.

**Supplementary Information:**

The online version contains supplementary material available at 10.1007/s00420-021-01717-8.

## Background

With falling birth rates and increasing life expectancy at higher ages, increasing the retirement age and getting more people to stay in the labour market at higher ages is a political priority in many countries in order to limit the dependency ratio—that is, the ratio of those who are not in the labour market to those who are. A high dependency ratio puts the working population under pressure, as each worker then has to support more non-workers (United Nations Department of Economic and Social Affairs 2020). Therefore, knowledge about factors that might promote or hinder people to remain in paid work in higher ages is warranted. Many studies have been conducted about associations between psychosocial work environment and future morbidity and mortality. However, so far research has mostly focused on work environment factors contributing to the intention to work after the statutory or traditional retirement age and has focused only to a lesser extent on factors contributing to actual remaining in paid work after the statutory or traditional retirement age (Browne et al. 2018).

In Sweden, the numbers and rates of people in paid work after the age of 65 have increased over the past decades, and there is a high rate of people in work at higher ages, compared to in other European countries, especially among women (Klevmarken 2010; OECD 2013).

Work-related characteristics are related to whether or not people retire early or late (Fisher et al. 2016). According to the theory first proposed by Karasek and Theorell, high demands in combinations with low control constitute a condition of “job strain” that is adverse for health (Karasek and Theorell 1990; Kristensen 1996). Thus, in this theory, high demands at work are not per se health hazardous. Instead, it is assumed that strain and health-hazardous psychological and psychophysiological processes are caused by the mismatch between high demands and low control, which consists of low decision authority and low skill discretion (Karasek and Theorell 1990; Kristensen 1996). On the other hand, those with not only a high level of work demands but also a high level of control might have more resources to cope with the level of demands, which may cause favourable reaction, such as the experience of success and mastery (Karasek and Theorell 1990; Kristensen 1996).

This would mean that people with jobs with high demands and low control would be more likely to withdraw from the labour market via sickness absence or disability pension (Williams-Whitt et al. 2015; Knardahl et al. 2017), and possibly also old-age pension, since that is sometimes taken for health reasons (van Rijn et al. 2014).

High psychosocial job demands have been identified as a factor in the decision to retire early (i.e., before the standard retirement age) (Fisher et al. 2016). Decision authority at work has also been identified as having an association with a longer time to exit work via old-age pension, disability pension, or unemployment after the age of 50 (Carr et al. 2016), and low control has been identified as being associated with disability pension after the age of 50 (Reinhardt et al. 2013).

However, while much research has supported this theorised association between high demands/low control and sickness absence/disability pension (Clausen et al. 2014; Janssens et al. 2014; Slany et al. 2014), the research concerning the role of demands has been more equivocal, especially regarding the association between high demands and sickness absence/disability pension (Rugulies et al. 2007; Jonge et al. 2010).

Although many studies have investigated the association between psychosocial working conditions and early exit from the labour market (Fisher et al. 2016; Knardahl et al. 2017), only a few studies have investigated this for remaining in work past the standard pension age. Having levels of job demands that correspond to levels of job control (i.e., high demands and high control, or low demands and low control), as well as having high work-time control have been shown to be associated with working beyond the standard pension age (Virtanen et al. 2014), whereas low job control has been shown to be associated with not working beyond the state pension age (Wahrendorf et al. 2017). However, a prospective study in Netherlands found that neither level of demands nor of control were associated with working beyond the standard pension age (de Wind et al. 2016).

These studies measured demands and control either at or just prior to the standard retirement age. However, it is possible that the influence of demands and control have a longer latency period, or that demands and control also are associated with labour market status after a longer follow-up time. Studies have shown associations between high job demands in mid-life, and poor health in older ages, for several different measures of health, including a higher risk of depression (Wahrendorf et al. 2013) and higher risk of musculoskeletal pain (Parker et al. 2013). Moreover, one study found a lower risk of serious health problems for men with high demands, but not for women (Nilsen et al. 2014). Low job control, in combination with either high or low job demands, is associated with a higher risk of depressive symptoms after retirement (Virtanen et al. 2015) and with a higher risk of serious health problems in old age for men, but not for women (Nilsen et al. 2014).

Given that research has shown associations between job demands and control in mid-life with health much later in life, and that research has shown associations between health in higher age and retiring/remaining in work (Nilsson 2016), it seems reasonable to expect there to be an association between job demands and control during the latter part of working life, and labour market status after the traditional retirement age. However, few studies have investigated this.

To the best of our knowledge, only two studies have investigated prospective associations of demands and control among older workers using several future labour market statuses as outcome in the same study, and both were conducted in populations below the standard retirement age even at the end of the follow-up. The first of these two studied specifically exit paths from paid work among older workers (unemployment, old-age pension, disability pension) with a 4-year follow-up (Robroek et al. 2013). This study found that low job control was associated with early exit from paid work via early old-age pension, unemployment, or disability pension, but that the association was strongest for disability pension (Robroek et al. 2013). High job demands in combination with low job control was also associated with work exit, although the strength of association was of similar magnitude as low control only (Robroek et al. 2013). The second study conducted by our research group on those who were aged 30–54 at baseline found that high demands were associated with a higher risk of being on old-age pension or emigrated, whereas low demands were associated with a higher risk of sickness absence/disability pension (Farrants et al. 2020). High control in combination with any level of demands was associated with lower risk of unemployment, and low demands in combination with low control was associated with a higher risk of sickness absence/disability pension even up to 11 years after exposure (Farrants et al. 2020). However, such long follow-up times are rare, and most studies of labour market status use a dichotomous working/retired measure, defined in various ways.

*The aim* of this study was to investigate associations between combinations of job demands and job control among people in paid work and aged 55–64 and their labour market status 11 years later.

## Methods

A population-based prospective cohort study was conducted.

### Data

We used anonymised microdata from the Longitudinal Integration Database for Health Insurance and Labour Market Studies (LISA) held by Statistics Sweden to identify the cohort and for information on age, sex, occupation, income from work and related benefits, country of birth, type of living area, family situation, educational level in 2001, number of net days with sickness absence/disability pension and unemployment benefits in 2001 and in 2012, income from old-age pension, as well as having emigrated or being dead by the end of 2012.

### Study population

Included were all those who were living in Sweden in December 2001, were aged 55–64 years, had an income from work or work-related benefits ≥ 8856 SEK (= 1003 Euros by the 2001 conversion rate), and had a registered occupation, using the Swedish Standard for Occupational Classification (SSYK by Swedish acronym). The income limit of 8856 SEK from work and/or work-related benefits was used to only include those with an income high enough to qualify for sickness absence benefits from the Swedish Social Insurance Agency. Both employees and self-employed persons were included. We excluded those who were on full-time disability pension for the whole of 2001, had full-time sickness absence for the whole of 2000 and 2001 (OECD 2010), or had early old-age pension. This gave a cohort of 616,818 individuals. We conducted an 11-year follow-up, at the year 2012.

### Variables

#### Exposure measures in 2001

For information on job demands and job control, we used the psychosocial job exposure matrix (JEM) from 1999, developed by Fredlund et al. (2000), where occupations were classified according to the Nordic Job Classification (NYK). For a job exposure matrix, using self-reported data, first an average group specific exposure value is calculated, based on many self-reports. This exposure value is subsequently assigned to all individuals according to job group, sex and age. The exposure value an individual is assigned is thus not based on the individual’s self-report but is based on the average level of all employees in the same job group.

The JEM we used includes specific values for job demands and job control for each Nordic Job Classification occupation, stratified for women and men and in three age groups: 16–29 years, 30–44 years, and 45–64 years, based on the mean score of women and men in the three respective age groups given in surveys (Fredlund et al. 2000). Supplementary table 1 shows the questions used to measure demands and control in the job exposure matrix (Fredlund et al. 2000).

Occupations with too few respondents were given values of neighbouring occupations. We translated the Swedish Standard for Occupational Classification categorisations of occupations into Nordic Job Classification to assign each individual a value for job demands and job control. For more information on this process, see Norberg et al. (2019).

### Outcome measures in 2012

We measured main labour market status in December 2012, in the following categories: long-term sickness absence (> 183 net days with sickness absence benefits from the Social Insurance Agency); no income/social assistance (no income from work, work-related benefits or pensions registered during 2012 or receipt of social assistance registered in 2012); old-age pension (more than half of yearly income from old-age pension); or in paid work (i.e., did not fulfil the criteria for long-term sickness absence, no income/social assistance, old-age pension, emigration, or death). We also included emigration and death as outcome categories, since they are potential competing outcomes that need to be handled in the analyses.

#### Covariates in 2001

Age: 55–59 or 60–64; sex: women or men; country of birth: Sweden, other Nordic country, other EU25, or rest of world (including missing); educational level: elementary (≤ 9 years or missing), high school (10–12 years), or university/college (> 12 years); type of living area: large city (Stockholm, Gothenburg, Malmö with surrounding municipalities), medium-sized town (municipalities with > 90,000 inhabitants within 30 km of municipal centre), or rural (municipalities with < 90,000 inhabitants within 30 km of municipal centre); family situation: married/cohabiting with children living at home, married/cohabiting with no children living at home, single with children living at home, or single/with no children living at home; unemployment or sickness absence/disability pension for more than half the year in 2001.

#### Analyses

We calculated descriptive statistics of the sociodemographic make-up of the cohort, as well as regarding their levels of job demands and job control according to the JEM, respectively. We used net days of sickness absence and disability pension. To calculate net days, part-time sickness absence/disability pension days were combined, e.g., two days of 50% sickness absence were combined to one net day.

We used multinomial logistic regression to calculate odds ratios (OR) with 95% confidence intervals (CI) of the association of job demands and job control with future paid work, long-term sickness absence/disability pension, no income/social assistance, old-age pension, emigration, and death, respectively, unadjusted and adjusted for all included sociodemographic covariates. We used old-age pension as the reference group.

To also capture those with more extreme values on demands and control than what is usually done in studies of psychosocial demands and control, we classified individuals in tertiles on both control and demands (Norberg et al. 2019) instead of in quartiles. This leads to the introduction of a model with nine groups based on their values on demands and control: (1) high demands/high control, (2) high demands/medium control, (3) high demands/low control, (4) medium demands/high control, (5) medium demands/medium control, (6) medium demands/low control, (7) low demands/high control, (8) low demands/medium control, and (9) low demands/low control. We used group (5) medium demands/medium control as the reference category for the analyses.

All analyses were run in the whole population as well as stratified by sex, as there was a significant interaction between sex and job demands/job control.

We also ran supplemental analyses to test for interactions between age group and job demands/control. Since the interaction effect showed to be significant (*p* < 0.001), we also ran analyses stratified by age group (55–59 and 60–64 in 2001, respectively), adjusting for age as a linear term. We ran further supplementary analyses where we included tertiles of job demands and job control separately, mutually adjusted and adjusted for all other factors in the analysis.

#### Social insurance system in Sweden

All people living in Sweden with income from work or unemployment benefits are from age 16 years covered by the public sickness absence insurance system and can be granted sickness absence benefits if their work capacity is reduced due to disease or injury. Day 1 is a waiting day, with 100% loss of income. After 7 days, a medical certificate is required. The employer reimburses income loss during days 2–14, after which sickness absence benefits are administered by the Swedish Social Insurance Agency. Sickness absence benefits can be granted for full time (100%) or part time (75, 50, or 25%) of ordinary work hours. Sickness absence benefits cover 80% of lost income, up to a certain level. After the age of 65, some restrictions apply. Individuals aged 65–69 years can get sickness absence benefits for up to a total of 180 days during those years, after which the Social Insurance Agency may restrict further claims if the reduced work capacity is assessed as permanent. From the age of 70, people may not claim sickness absence benefits for more than 180 days.

All people in Sweden aged 19–65 can claim disability pension if their work capacity is reduced due to disease or injury. Disability pension can also be granted for full- or part time and cover about 64%, up to a certain level, of lost income.

From age 65, it is no longer possible to have disability pension or unemployment benefits. The retirement age in Sweden is flexible. In the time period of this study, old-age pension could be claimed from age 61, for part- or full time, although 65 was still the most common age of retirement. Individuals had the right to keep their permanent position up to age 67, after which the decision to retain them or terminate their employment was up to their employer.

## Results

Table 1 shows the sociodemographic characteristics of the cohort. The majority were born in Sweden, and had no sickness absence/disability pension or unemployment at baseline. Approximately half were married/cohabiting without children < 18 years living at home. The distribution of sociodemographic factors varied somewhat between those who were aged 55–59 and those who were aged 60–64 at baseline. There were far fewer who were aged 60–64 (192,730 people) than who were aged 54–59 (424,088). A somewhat higher proportion of those aged 60–64 had only elementary education than among those initially aged 55–59. A smaller proportion of those aged 60–64 had children living at home, either as singles or as married/cohabiting (13.52 vs. 25.25%).

**Table 1:** Sociodemographic background of the cohort of women and men in Sweden in 2001 who had income from work and were aged 55–64 (*n* = 616,818)

All	All		Women		Men	
	*n*	%	*n*	%	*n*	%
Total	616,818		313,100		303,718	
Sex						
Women	313,100	50.76				
Men	303,718	49.24				
Birth country						
Sweden	563,710	91.39	286,882	91.63	276,828	91.15
Other Nordic country	28,109	4.56	15,825	5.05	12,284	4.04
EU25 without Nordic countries	14,409	2.34	6388	2.04	8021	2.64
Rest of the world	10,590	1.72	4005	1.28	6585	2.17
Type of living area						
Big cities	209,911	34.03	109,298	34.91	100,613	33.13
Medium-sized cities	220,347	35.72	110,480	35.29	109,867	36.17
Small cities	186,560	30.25	93,322	29.81	93,238	30.7
Education						
Elementary (0–9 years)	181,248	29.38	81,793	26.12	99,455	32.75
High school (10–12 years)	255,566	41.43	133,401	42.61	122,165	40.22
University/college (> 12 years)	180,004	29.18	97,906	31.27	82,098	27.03
Family situation						
Married/living with partner without children living at home	314,292	50.95	163,972	52.37	150,320	49.49
Married/living with partner with children living at home	111,394	18.06	42,506	13.58	68,888	22.68
Single/divorced/separated/widowed without children living at home	169,397	27.46	91,582	29.25	77,815	25.62
Single/divorced/separated/widowed with children living at home	21,735	3.52	15,040	4.8	6695	2.2
Unemployment or sickness absence/disability pension in 2001						
No long-term sickness absence/disability pension or unemployment	564,581	91.53	282,869	90.34	281,712	92.75
Unemployed > 183 days	17,727	2.87	6778	2.16	10,949	3.6
Sickness absence/disability pension > 183 net days	34,510	5.59	23,453	7.49	11,057	3.64

54–59	*n*	%	*n*	%	*n*	%
Total	424,088		214,392		209,696	
Sex						
Women	214,392	50.55				
Men	209,696	49.45				
Birth country						
Sweden	389,755	91.9	197,265	92.01	192,490	91.79
Other Nordic country	18,249	4.3	10,337	4.82	7912	3.77
EU25 without Nordic countries	8,678	2.05	3881	1.81	4797	2.29
Rest of the world	7,406	1.75	2909	1.36	4497	2.14
Type of living area						
Big cities	145,293	34.26	75,513	35.22	69,780	33.28
Medium-sized cities	151,284	35.67	75,807	35.36	75,477	35.99
Small cities	127,511	30.07	63,072	29.42	64,439	30.73
Education						
Elementary (0–9 years)	114, 674	27.04	51,016	23.8	63,658	30.36
High school (10–12 years)	181,342	42.76	94,019	43.85	87,323	41.64
University/college (> 12 years)	128,072	30.2	69,357	32.35	58,715	28.00
Family situation						
Married/living with partner without children living at home	203,810	48.06	108,739	50.72	95,071	45.34
Married/living with partner with children living at home	90,069	21.24	35,152	16.4	54,917	26.19
Single/divorced/separated/widowed without children living at home	113,192	26.69	58,718	27.39	54,474	25.98
Single/divorced/separated/widowed with children living at home	17,017	4.01	11,783	5.5	5234	2.5
Unemployment or sickness absence/disability pension in 2001						
No long-term sickness absence/disability pension or unemployment	392,103	92.46	195,634	91.25	196,469	93.69
Unemployed > 183 days	11,032	2.6	4246	1.98	6786	3.24
Sickness absence/disability pension > 183 net days	20,953	4.94	14,512	6.77	6441	3.07

60–64	*n*	*%*	*n*	*%*	*n*	*%*
Total	92,730		98,708		94,022	
Sex						
Women	98,708	51.22				
Men	94,022	48.78				
Birth country						
Sweden	173,955	90.26	89,617	90.79	84,338	89.7
Other Nordic country	9860	5.12	5488	5.56	4372	4.65
EU25 without Nordic countries	5731	2.97	2507	2.54	3224	3.43
Rest of the world	3184	1.65	1096	1.11	2088	2.22
Type of living area						
Big cities	64,618	33.53	33,785	34.23	30,833	32.79
Medium-sized cities	69,063	35.83	34 673	35.13	34,390	36.58
Small cities	59,049	30.64	30 250	30.65	28,799	30.63
Education						
Elementary (0–9 years)	66,574	34.54	30,777	31.18	35,797	38.07
High school (10–12 years)	74,224	38.51	39,382	39.9	34,842	37.06
University/college (> 12 years)	51,932	26.95	28,549	28.92	23,383	24.87
Family situation						
Married/living with partner without children living at home	110,482	57.32	55,233	55.96	55,249	58.76
Married/living with partner with children living at home	21,325	11.06	7354	7.45	13,971	14.86
Single/divorced/separated/widowed without children living at home	56,205	29.16	32,864	33.29	23,341	24.83
Single/divorced/separated/widowed with children living at home	4718	2.45	3257	3.30	1461	1.55
Unemployment or sickness absence/disability pension in 2001						
No long-term sickness absence/disability pension or unemployment	172,478	89.49	87,235	88.38	85,243	90.66
Unemployed > 183 days	6695	3.47	2532	2.57	4163	4.43
Sickness absence/disability pension > 183 net days	13,557	7.03	8941	9.06	4616	4.91

**Fig. 1:**
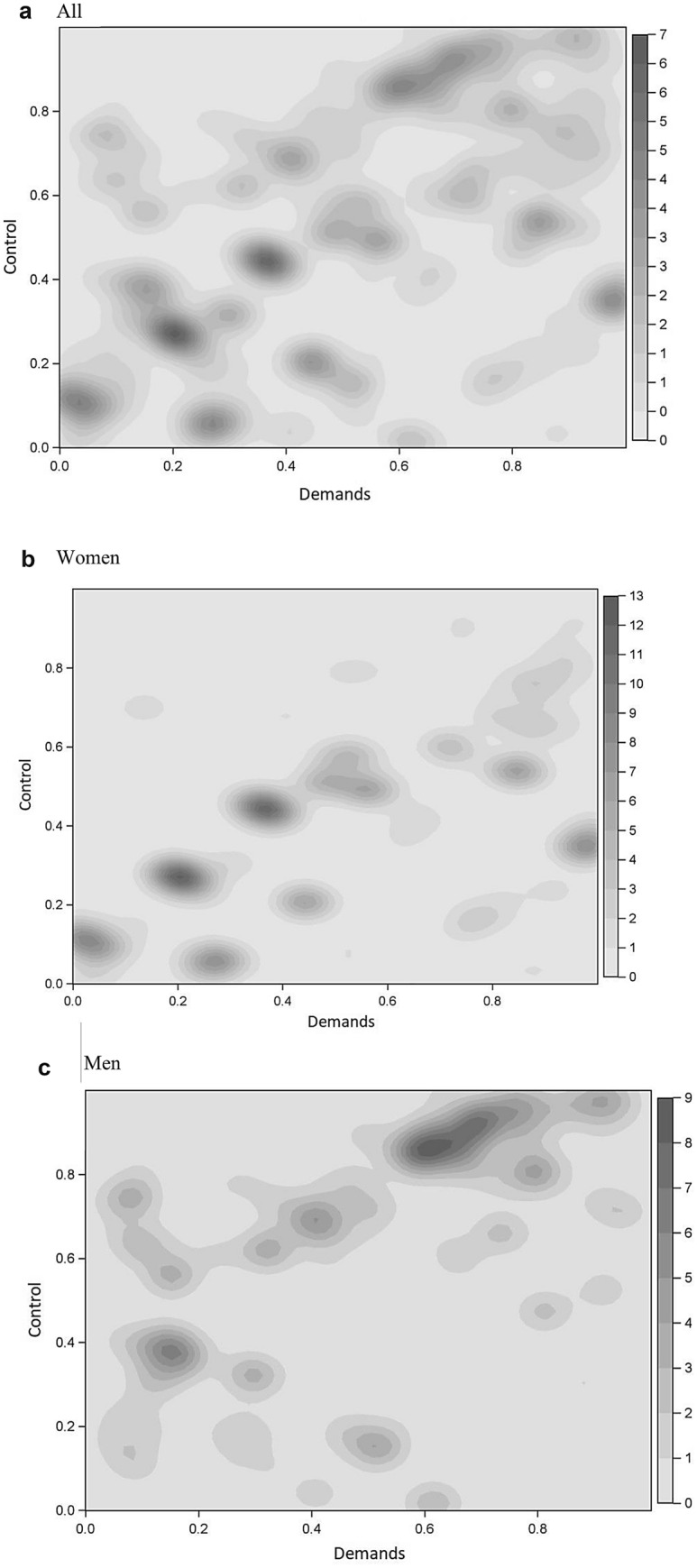
Kernel density plots over Job Exposure Matrix values of individuals for job demands and job control among **a** all individuals among all aged 55–64 in paid work in Sweden 2001 (*n* = 616,818) and **b** women (*n* = 313,100) and men (*n* = 303,718), respectively

Figure 1 shows kernel density plots over the distribution of demands and control in the cohort, where darker areas indicate a higher concentration of individuals. The highest concentration of women was low demands and low control, and the level of job control seemed to increase linearly with the level of demands for many women. For men, the highest concentration was high demands and high control. Unlike women, there was a relatively high concentration of men who had low demands and medium to high control. Supplementary Fig. 1 shows kernel density plots over the distribution of job demands and control, stratified by age group in 2001, 55–59 or 60–64. There were no major differences in the distribution of job demands and control by age group.

**Fig. 2:**
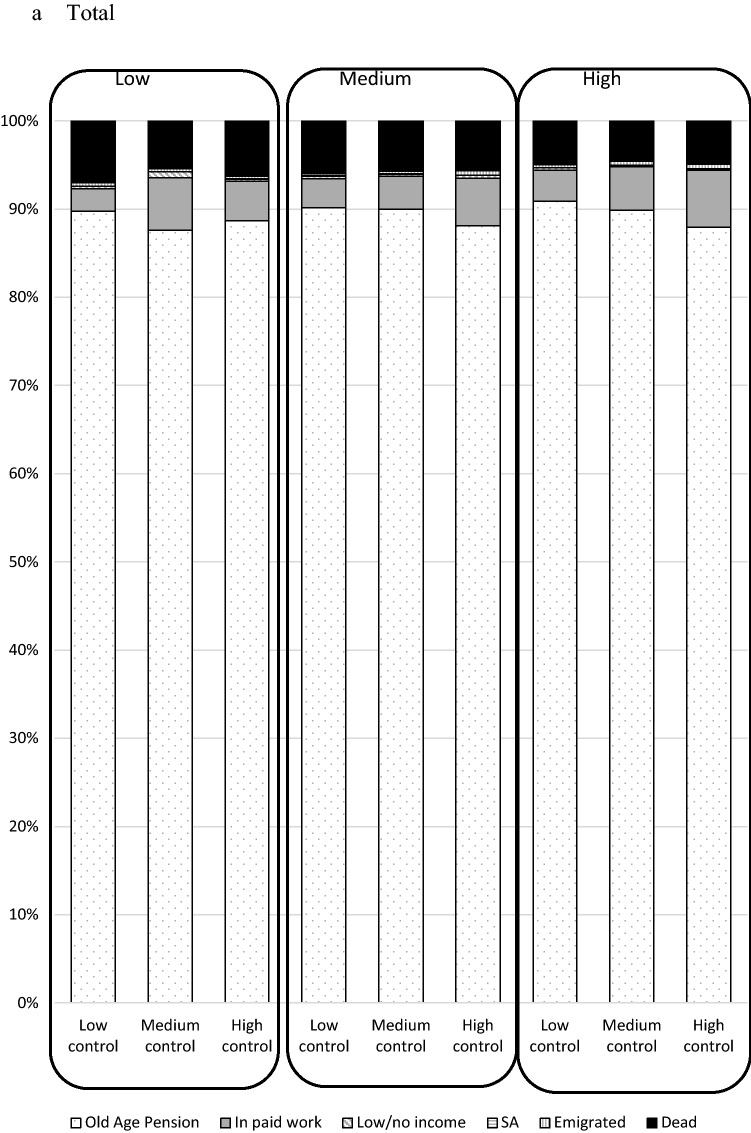

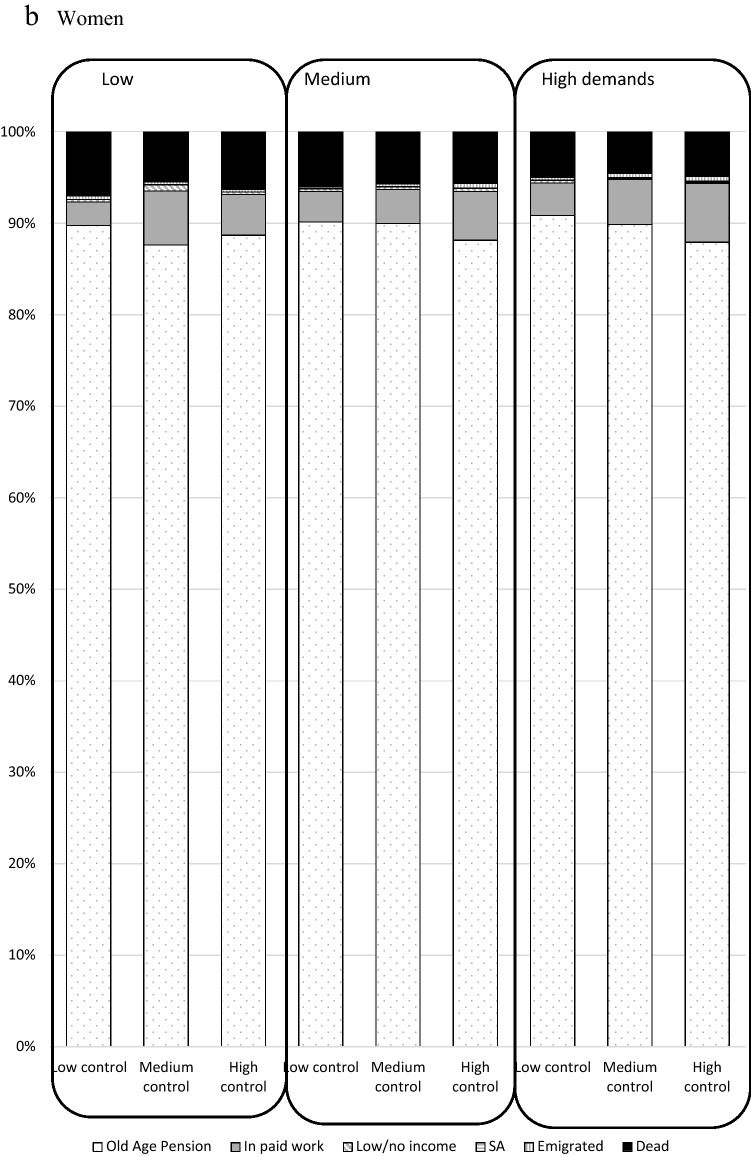

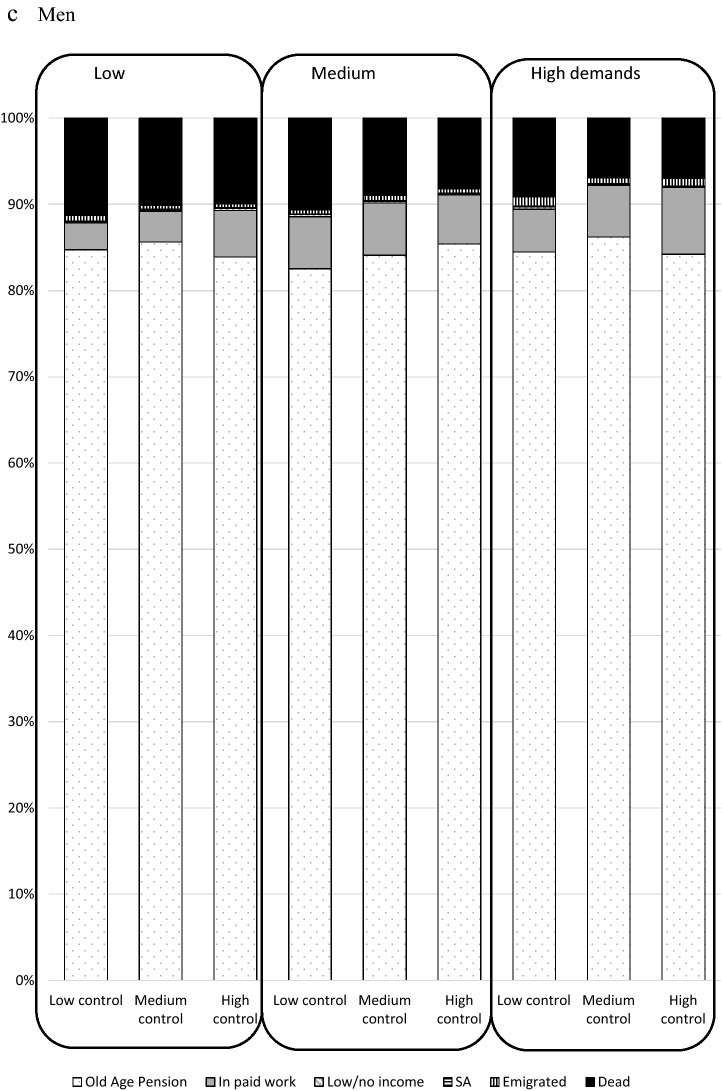
Labour market status in 2012 by job demands and job control in 2001 among all in paid work in Sweden in 2001 and aged 55–64 (*N* = 616,818), total and stratified by sex

Figure 2 shows the prevalence (%) of each labour market status in 2012, by combinations of job demands/control in 2001. The vast majority (over 85%) in each group of demands/control were old-age pensioned in 2012. A slightly higher proportion of women than men were old-age pensioned in each group of demands/control, and a slightly higher proportion of men than women were dead in each group of demands/control. Supplementary Fig. 2 shows that a smaller proportion of those who were 60–64 in 2001 were in paid work and more had died than those who were 55–59 at inclusion in 2001. A larger proportion of those who were aged 60–64 were in old-age pension in 2001, in all categories of demands and control.

This is also supported by the results in Supplementary Fig. 3, which shows proportions with each labour market status in 2012 by individual years of age in 2012.

Table 2 shows ORs from the multinomial logistic regression, with old-aged pension as the reference group for the outcome, and medium demands/medium control as the reference group for the exposure, unadjusted and adjusted for sociodemographic characteristics.

**Table 2:** Odds ratios (OR) and 95% confidence intervals (CI) of labour market status (reference group = old-age pension, total = 537,882, women = 280,631, men = 257,251) in 2012 by combinations of job demands/control (reference group = medium demands/medium control) in 2001, among 616,818 individuals (313,100 women and 303,718 men) aged 55–64 in 2001 with income from work, crude and adjusted

	In paid work (*n* = 29,210)		No income/social assistance (*n *= 1258)		Sickness absence/disability pension (*n* = 162)		Emigrated (*n* = 3133)		Dead (*n* = 45,173)	
	Crude OR (95% CI)	Adjusted OR (95% CI)	Crude OR (95% CI)	Adjusted OR (95% CI)	Crude OR (95% CI)	Adjusted OR (95% CI)	Crude OR (95% CI)	Adjusted OR (95% CI)	Crude OR (95% CI)	Adjusted OR (95% CI)
Total										
Low demands/ low control	0.68 (0.65–0.72)	0.74 (0.71–0.78)	0.96 (0.81–1.14)	1.04 (0.88–1.25)	0.65 (0.35–1.21)	0.70 (0.37–1.32)	1.24 (1.08–1.42)	1.05 (0.86–1.27)	1.34 (1.30–1.39)	1.16 (1.11–1.21)
Low demands/ medium control	0.94 (0.89–1.00)	0.79 (0.74–0.84)	0.72 (0.57–0.92)	0.92 (0.70–1.20)	1.36 (0.71–2.63)	1.00 (0.48–2.06)	1.41 (1.19–1.67)	0.88 (0.69–1.12)	1.69 (1.62–1.75)	1.05 (0.99–1.11)
Low demands/ high control	1.34 (1.26–1.43)	1.11 (1.06–1.19)	1.01 (0.76–1.34)	1.11 (0.83–1.49)	1.45 (0.64–3.30)	1.13 (0.48–2.66)	1.22 (0.97–1.53)	0.83 (0.61–1.13)	1.55 (1.47–1.63)	1.08 (1.03–1.16)
Medium demands/ low control	1.25 (1.18–1.32)	1.16 (1.10–1.23)	1.01 (0.82–1.26)	1.18 (0.94–1.48)	1.78 (0.96–1.31)	1.49 (0.78–2.85)	1.13 (0.94–1.36)	0.88 (0.69–1.13)	1.45 (1.39–1.51)	1.09 (1.03–1.15)
Medium demands/medium control	1	1	1	1	1	1	1	1	1	1
Medium demands/ high control	1.46 (1.39–1.53)	1.12 (1.06–1.18)	0.66 (0.53–0.82)	0.86 (0.67–1.10)	1.21 (0.65–2.56)	0.89 (0.45–1.76)	1.49 (1.28–1.73)	1.05 (0.84–1.31)	1.33 (1.27–1.39)	0.98 (0.93–1.03)
High demands/ low control	0.96 (0.89–1.03)	0.81 (0.75–0.87)	0.73 (0.54–0.99)	0.71 (0.52–0.96)	1.41 (0.64–3.11)	1.25 (0.57–2.78)	1.44 (1.17–1.76)	1.10 (0.83–1.44)	0.97 (0.91–1.02)	0.96 (0.90–1.03)
High demands/ medium control	1.29 (1.23–1.36)	0.93 (0.89–0.98)	0.67 (0.45–0.72)	0.52 (0.41–0.67)	1.05 (0.55–2.00)	0.91 (0.45–1.80)	1.47 (1.26–1.71)	1.10 (0.88–1.36)	0.84 (0.80–0.88)	0.91 (0.86–0.96)
High demands/ high control	1.92 (1.84–2.00)	1.21 (1.18–1.33)	0.53 (0.43–0.65)	0.59 (0.47–0.75)	1.30 (0.75–2.28)	0.88 (0.47–1.64)	2.42 (2.12–2.76)	1.45 (1.19–1.77)	1.08 (1.05–1.13)	0.92 (0.87–0.96)

Women	In paid work (*n* = 12,249)		No income/social assistance (*n* = 762)		Sickness absence/disability pension (*n* = 64)		Emigrated (*n* = 1173)		Dead (*n* = 18,221)	
Low demands/low control	0.69 (0.66–0.73)	0.83 (0.79–0.88)	1.06 (0.88–1.27)	1.09 (0.90–1.32)	0.56 (0.26–1.23)	0.76 (0.34–1.71)	1.16 (0.98–1.37)	1.02 (0.80–1.31)	1.24 (1.19–1.29)	1.17 (1.11–1.23)
Low demands/medium control	1.63 (1.33–1.99)	1.49 (1.21–1.83)	2.59 (1.44–4.65)	2.40 (1.34–4.33)	Too few	Too few	1.13 (0.50–2.54)	1.60 (0.59–4.37)	0.98 (0.80–1.21)	1.13 (0.90–1.46)
Low demands/high control	1.22 (1.07–1.39)	1.21 (1.06–1.38)	0.97 (0.57–1.63)	0.97 (0.57–1.64)	0.85 (0.11–6.48)	0.85 (0.11–6.46)	0.95 (0.58–1.56)	1.11 (0.59–2.06)	1.12 (1.00–1.25)	1.18 (1.03–1.34)
Medium demands/low control	0.90 (0.82–0.97)	0.96 (0.89–1.05)	0.96 (0.71–1.29)	0.98 (0.72–1.32)	1.48 (0.57–3.86)	1.48 (0.57–3.87)	0.77 (0.57–1.05)	0.90 (0.61–1.34)	1.05 (0.98–1.12)	1.04 (0.96–1.13)
Medium demands/medium control	1	1	1	1	1	1	1	1	1	1
Medium demands/high control	1.47 (1.34–1.61)	1.32 (1.20–1.45)	1.29 (0.90–1.83)	1.27 (0.88–1.81)	1.67 (0.55–5.06)	1.68 (0.55–5.10)	1.59 (1.17–2.16)	1.65 (1.10–2.46)	1.01 (0.93–1.11)	1.02 (0.91–1.14)
High demands/low control	0.94 (0.87–1.03)	0.82 (9.75–0.89)	0.61 (0.42–8.88)	0.59 (0.41–0.86)	1.21 (0.46–3.17)	1.21 (0.46–3.18)	1.06 (0.81–1.39)	1.05 (0.71–1.54)	0.86 (0.80–0.92)	0.92 (0.85–1.01)
High demands/medium control	1.33 (1.26–1.41)	0.94 (0.88–1.00)	0.56 (0.43–0.72)	0.51 (0.38–0.69)	0.74 (0.31–1.73)	0.74 (0.32–1.72)	1.49 (1.25–1.79)	1.31 (1.00–1.74)	0.80 (0.76–0.84)	0.94 (0.87–1.01)
High demands/high control	1.82 (1.71–1.93)	1.27 (1.19–1.36)	0.73 (0.55–0.97)	0.67 (0.49–0.91)	0.87 (0.36–2.13)	0.90 (0.37–2.18)	1.78 (1.46–2.17)	1.69 (1.19–2.13)	0.89 (0.84–0.95)	1.01 (0.94–1.09)

Men	In paid work (*n* = 16,931)		No income/social assistance (*n* = 496)		Sickness absence/disability pension (*n* = 98)		Emigrated (*n* = 1960)		Dead (*n* = 26,952)	
Low demands/low control	0.52 (0.47–0.57)	0.57 (0.51–0.63)	0.71 (0.45–1.13)	0.71 (0.45–1.14)	0.57 (0.19–1.69)	0.57 (0.19–1.71)	1.00 (0.76–1.30)	0.91 (0.64–1.29)	1.24 (1.16–1.34)	1.12 (1.03–1.23)
Low demands/medium control	0.58 (0.53–0.64)	0.64 (0.59–0.71)	0.70 (0.45–1.10)	0.67 (0.43–1.05)	0.71 (0.26–1.95)	0.68 (0.25–1.88)	0.76 (0.58–0.98)	0.72 (0.51–1.02)	1.11 (1.03–1.19)	1.01 (0.93–1.11)
Low demands/high control	0.90 (0.81- 0.99)	0.93 (0.84–1.03)	1.11 (0.69–1.79)	0.89 (0.55–1.44)	0.86 (0.27–2.70)	0.87 (0.27–2.73)	0.71 (0.52–0.98)	0.66 (0.44–0.99)	1.11 (1.03–1.21)	1.03 (0.94–1.14)
Medium demands/low control	1.02 (0.93–1.12)	1.09 (0.99–1.20)	1.15 (0.73–1.80)	1.17 (0.74–1.85)	1.14 (0.42–3.26)	1.07 (0.38–3.00)	0.81 (0.61–1.08)	0.75 (0.51–1.09)	1.20 (1.12–1.30)	1.09 (0.99–1.20)
Medium demands/medium control	1	1	1	1	1	1	1	1	1	1
Medium demands/high control	0.93 (0.87–1.02)	0.93 (0.85–1.01)	0.59 (0.38–0.92)	0.63 (0.40–0.97)	0.58 (0.21–1.28)	0.58 (0.21–1.57)	0.80 (0.61–1.03)	0.79 (0.57–1.11)	0.90 (0.94–0.97)	0.95 (0.88–1.04)
High demands/low control	0.82 (0.71–0.94)	0.72 (0.62–0.83)	1.24 (0.68–2.27)	0.97 (0.53–1.78)	1.08 (0.26–4.50)	1.04 (0.25–4.37)	1.72 (1.23–2.40)	1.08 (0.71–1.65)	1.02 (0.91–1.13)	1.01 (0.89–1.15)
High demands/medium control	0.97 (0.88–1.07)	0.80 (0.72–0.88)	0.63 (0.36–1.08)	0.54 (0.31–0.95)	1.08 (.26–4.50)	0.69 (0.20–2.41)	1.05 (0.78–91.42)	0.73 (0.49–1.09)	0.75 (0.69–0.82)	0.87 (0.78–0.96)
High demands/high control	1.28 (1.81–1.39)	1.10 (1.01–1.20)	0.47 (0.32–0.74)	0.46 (0.30–0.72)	0.68 (0.26–1.80)	0.65 (0.25–1.73)	1.48 (1.16–1.89)	1.17 (0.85–1.61)	0.78 (0.72–0.83)	0.88 (0.80–0.96)

Adjusted for age, birth country, type of living area, family situation, educational level, labour market status in 2001

In the fully adjusted model, those with high demands and high control were more likely to be in paid work (OR women 1.27 (CI 1.19–1.36), OR men 1.10 (CI 1.01–1.20)). For women, high control, regardless of level of demands, was associated with being in paid work (OR medium demands/high control 1.32 (CI 1.20–1.45); OR low demands/high control 1.21 (CI 1.06–1.38)); however, this was not the case for men. Both men and women with low demands and low control (OR women 0.83 (CI 0.79–0.88); OR men 0.57 (CI 0.51–0.63)), as well as men with low demands and medium control (OR 0.64 (CI 0.59–0.71)) were less likely to be in paid work.

Both women and men with high demands and high or medium control were less likely to have no income/social assistance (OR women high demands/high control 0.67 (CI 0.49–0.91), high demands/medium control 0.51 (0.38–0.69); OR men high demands/high control 0.46 (CI 0.30–0.72), high demands/medium control 0.54 (CI 0.31–0.95)). This was also the case for women with high demands/low control (OR 0.59 (CI 0.41–0.86)), whereas the association for men was not statistically significant.

None of the associations between job demands/job control and long-term sickness absence (> 183 days) were statistically significant.

Women with high demands and high control were more likely to have emigrated (OR women 1.69 (OR 1.19–2.13)), as were women with medium demands and high control (OR 1.31 (CI 1.00–1.74)). Men with low demands and high control were less likely to have emigrated (OR 0.66 (CI 0.44–0.99)).

Men with high demands and high or medium control (OR high demands/high control 0.88 (CI 0.80–0.96), high demands/medium control 0.87 (CI 0.78–0.96)) were less likely to have died at end of follow-up. Women with low demands and high or low control (OR low demands/high control 1.18 (CI 1.03–1.34), low demands/low control 1.17 (CI 1.11–1.23)), and men with low demands and low control (OR 1.12 (CI 1.03–1.23)) were slightly more likely to have died at the end of follow-up.

Supplementary Table 2 shows odds ratios for the association between labour market state at follow-up by demands/control at baseline, among those who were 54–59 and 60–64 at baseline, respectively. This table shows that most of these associations were in the same direction in both the two age groups, although they were occasionally of different magnitudes and a few statistically significant associations in the whole population became non-significant, especially in those who were aged 60–64 in 2001, potentially due to the smaller size of the group.

Supplementary table 3 shows that when considered separately, both high demands and high control were associated with greater odds of being in paid work—however, this effect became non-significant for demands when adjusting for control and sociodemographic covariates. High control remained a predictor of being in paid work, even when controlling for demands and sociodemographic factors, although slightly attenuated. Both high demands and high control were associated with a lower risk of having no income/social assistance, but in the adjusted model, only the estimate for demands remained statistically significant.

## Discussion

### Summary of results

In this prospective, population-based cohort study, we found that most of the 616,818 individuals aged 55–64 and in paid work in 2001 were on old-age pension 11 years later. Job demands and job control in 2001 were associated with labour market status in 2012. High control regardless of demands was associated with being in paid work for women; whereas for men, high demands in combination with high control were associated with being in paid work. Low demands in combination with low control were associated with a lower likelihood of being in paid work for both women and men. High demands were associated with a lower likelihood of having no income/social assistance, and a higher likelihood of being emigrated.

### Discussion of results

The theory developed by Karasek and Theorell identified jobs with high demands and low control as particularly adverse for health outcomes (Karasek and Theorell 1990). However, we did not identify such an effect in our results regarding future labour market situation among workers of higher ages. Those in jobs with high demands/low control at inclusion were less likely to have no income/social assistance (although this was statistically significant in women only), and less likely to be dead at follow-up (again, statistically significant in women only) than those with medium demands/medium control.

On the other hand, those with low demands/low control were less likely to still be in paid work than those with medium demands/medium control, but since those with high demands and low control were also less likely to be in paid work, it is possible that for being in paid work, it is the level of control that is most important, rather than the combinations of demands and control. This is further supported by our finding that high control in all combinations of demands among women was associated with remaining in paid work, although for men this was only the case in combination with high demands, as well as our finding that when considered separately, high control was associated with higher odds of being in paid work when adjusted for demands and sociodemographic factors, but high demands were not significantly associated with remaining in paid work in the adjusted model. Previous literature has also identified job control and decision authority as important factors for intending to or actually staying in work at higher ages (Virtanen et al. 2014; Carr et al. 2016; Fisher et al. 2016; Knardahl et al. 2017; Wahrendorf et al. 2017), including one systematic review (Browne et al. 2018), giving further support to the importance of control.

The gender differences in the results, with high control being associated with a higher likelihood of remaining in paid work regardless of the level of job demands for women, but not for men, indicate that there are differential effects on the importance of job control for women and men in relation to remaining in paid work. The gender-segregation of occupations is very high in Sweden, with many jobs being numerically either female- or male dominated (Gonäs et al. 2019a, b). It could be that the differences we found are due to women and men being in different types of jobs, even with the same level of demands and control. It could also be due to differences in other factors that influence employment among women and men, such as family-work interference, income, or health and morbidity (Blackburn et al. 2016) or social support at work (Viswesvaran et al. 1999).

We did not find any such clear patterns regarding job demands and the association with being in paid work. A review of the literature regarding associations between psychosocial work characteristics and retirement intentions and retirement behaviour found that there was only very limited evidence for an association between job demands and retirement (Browne et al. 2018).

Previous research has found that those who remain in paid work after age 65 are more likely to be self-employed (Wahrendorf et al. 2017; Delegationen för senior arbetskraft 2020). It may be that those occupations that have a higher level of job demands and control are also those that have a higher proportion of self-employed, and that it is this that is the driving factor, rather than the actual level of demands and control. This should be investigated further.

The odds ratios for death were small, rarely below 0.85 or above 1.15, despite death being the second-most frequent outcome in the cohort, after old-age retirement. While several studies have found associations between combinations of job demands/control and mortality (especially cardiovascular mortality, but also mortality from certain cancers) (Kivimäki et al. 2002, 2018; Kuper and Marmot 2003; Tobiasz-Adamczyk et al. 2013; Gonzalez-Mulé and Cockburn 2017), others have found no such associations (Sabbath et al. 2015; Trudel-Fitzgerald et al. 2017). Separate associations between high demands (Kuper and Marmot 2003; Sabbath et al. 2015) as well as low control (Johnson et al. 1996; Kivimäki et al. 2002) with mortality have also been found in some studies. On the other hand, others have found no such associations between high demands (Johnson et al. 1996; Kivimäki et al. 2002; Shirom et al. 2011) or low control (Kuper and Marmot 2003; Shirom et al. 2011) and mortality. An overview of systematic reviews concluded that high demands and low control were associated with cardiovascular mortality and morbidity, especially for men, although there is no agreement on whether high demands, low control, or the combination is the most salient factor in the association (Fishta and Backé 2015). In our study, we have a quite strong health selection effect, in that we only study those who still were in paid work in the ages 55–64 at baseline in 2001—those who had been granted disability pension or taken out early old-age pension before the age of 65 were not included. This may partially contribute to the low differences between the exposure groups regarding the risk of death. They may also be due to the use of a job exposure matrix rather than self-report, as there may be reverse causation where those with poorer health report their demands and control as worse than those in better health (Kolstad et al. 2011), leading to associations seeming stronger than they actually are. It may also be due to the use of the group with medium demands and control as the reference group, whereas other studies tend to use either the lowest or the highest group, based on quartiles. Further, while the use of a job exposure matrix can overcome the problems of reverse causation regarding morbidity, not everyone in the same occupation experiences the same level of demands and control. How people experience demands and control is also dependent on personal and contextual factors (Bambra et al. 2007). It may be that this experience of demands and control has an association with morbidity beyond that of the occupation aggregated measures of demands and control, and it is that additional effect that is picked up by studies using self-reported information.

The job demands–control theory also posits that high demands/high control jobs may be protective for health. Although this has been somewhat neglected in research compared to high demands/low control jobs as a risk factor, it has been supported regarding cognitive decline (Pan et al. 2019) and mortality (Gonzalez-Mulé and Cockburn 2017). It has also been supported regarding a lower risk of sickness absence/disability pension and unemployment among those below 65 years at end of follow-up, although more strongly for men than for women (Norberg et al. 2019). Our study offers additional support to this hypothesised protective effect of jobs with high demands and high control, as individuals with high demands and high control were more likely to still be in paid work and less likely to have no income/social assistance. Men with high demands and high or medium control were also less likely to have died. This may also partially be due to jobs with high demands and high control often being better paid than those with low demands and low control (Karasek 1979), and previous research has shown that those with high incomes may be more likely to remain in paid work (Fisher et al. 2016).

No significant associations between job demands/control and future sickness absence were found, likely owing to the low numbers with that outcome. Previous research investigating sickness absence after the age of 65 in Sweden found that despite increasing rates of people being in paid work after 65, rates of sickness absence were not increasing (Farrants et al. 2017).

We have conducted another study using the same methods, but on a younger cohort (below 65 at the end of follow-up), and found similar results as in this study—especially regarding the association between high demands/high control and likelihood of remaining in paid work relative to other labour market states (Farrants et al. 2020).

However, in that previous study, we found that a combination of high demands and high control were associated with a higher likelihood of being on (early) old-age pension, which is to some extent contradicted by our results here, where high demands and high control were associated with a higher likelihood of being in paid work relative to being on old-age pension. High demands and high control seem, thus, to be a predictor both of early old-age pension and of being in paid work past “normal” pension age. This shows that the interaction between job demands and control and working and retirement in later working life is not simple, and warrants further research.

### Strengths and limitations

The main strengths of this study are the very large study population, the coverage of the entire working population rather than a selected sample, also allowing for sub-group analyses, the use of high-quality register data (Ludvigsson et al. 2019) rather than self-reports for assessing the outcomes, and the prospective cohort design without loss to follow-up, and the long follow-up period (11 years). Another strength is that we could use a JEM (Fredlund et al. 2000) to measure job demands and job control, meaning that the analyses was not vulnerable to recall and reporting bias. This is important, because self-reported data on job demands and job control might be influenced by morbidity (Kolstad et al. 2011), which is related to the outcomes in this study, and may, thus, lead to reverse causation.

There are also certain limitations to this study. We had no information about how long the people in the cohort had been exposed to the job demands/control at inclusion or whether they worked full- or part time, nor about job demands/control or labour market status in the 11 years between baseline measures in 2001 and the outcome. We also did not adjust for self-employment in this study: those who are self-employed may be in occupations with higher demands and control (although not necessarily), and are more likely to remain in paid work after age 65. Further, we had no information on sickness absence-spells < 15 days, and information on occupation was not updated every year for every individual, thus, some individuals might have had another occupation in 2001 than the one registered. Furthermore, the use of the JEM meant that we cannot distinguish individual differences in demands and control between those who have been assigned the same value based on their age, sex and occupation. There may also be selection effects in the kinds of work people do—in that individuals with certain kinds of morbidity may have transferred into jobs with different levels of demands and control, or even left the labour market before age 54.

### Conclusion

Our study shows that there is an association between job demands/job control when aged 55–64 and labour market status 11 years later. High demands and high control were both associated with still being in paid work, whereas low demands and low control, especially in combination, were associated with a higher risk of death or having no income/social assistance. These results support the theory that high demand/high control jobs are beneficial not only for health but also for future labour market status.

## Supplementary Information

Below is the link to the electronic supplementary material.

Supplementary file1 (PDF 1402 kb)

## Data Availability

These data cannot be made publically available due to privacy regulations. According to the General Data Protection Regulation, the Swedish law SFS 2018:218, the Swedish Data Protection Act, the Swedish Ethical Review Act, and the Public Access to Information and Secrecy Act, these type of sensitive data can only be made available for specific purposes, including research, that meets the criteria for access to this type of sensitive and confidential data as determined by a legal review. Readers may contact Professor Kristina Alexanderson (kristina.alexanderson@ki.se) regarding the data.
