# Supplementary material for: Associations between combinations of job demands and job control among 6,16,818 people aged 55–64 in paid work with their labour market status 11 years later: a prospective cohort study
Source: Int Arch Occup Environ Health. 2021 Jun 7;95(1):169–85. doi: 10.1007/s00420-021-01717-8 (PMC8755665; doi:10.1007/s00420-021-01717-8)
Supplement: Supplementary file 1 — Supplementary file1 (PDF 1402 kb) [file 420_2021_1717_MOESM1_ESM.pdf]

Farrants K, Head J, Framke E, Rugulies R, Alexanderson K. *Associations between combinations of job demands and job control among 616,818 people aged 55-64 in paid work with their labour market status 11 years later: a prospective cohort study.* Supplementary material.

**Supplementary material.**

**Supplementary Figure 1. Kernel density plots over Job Exposure Matrix values of individuals for job demands and job control stratified by age group in 2001.**

a) 55-59

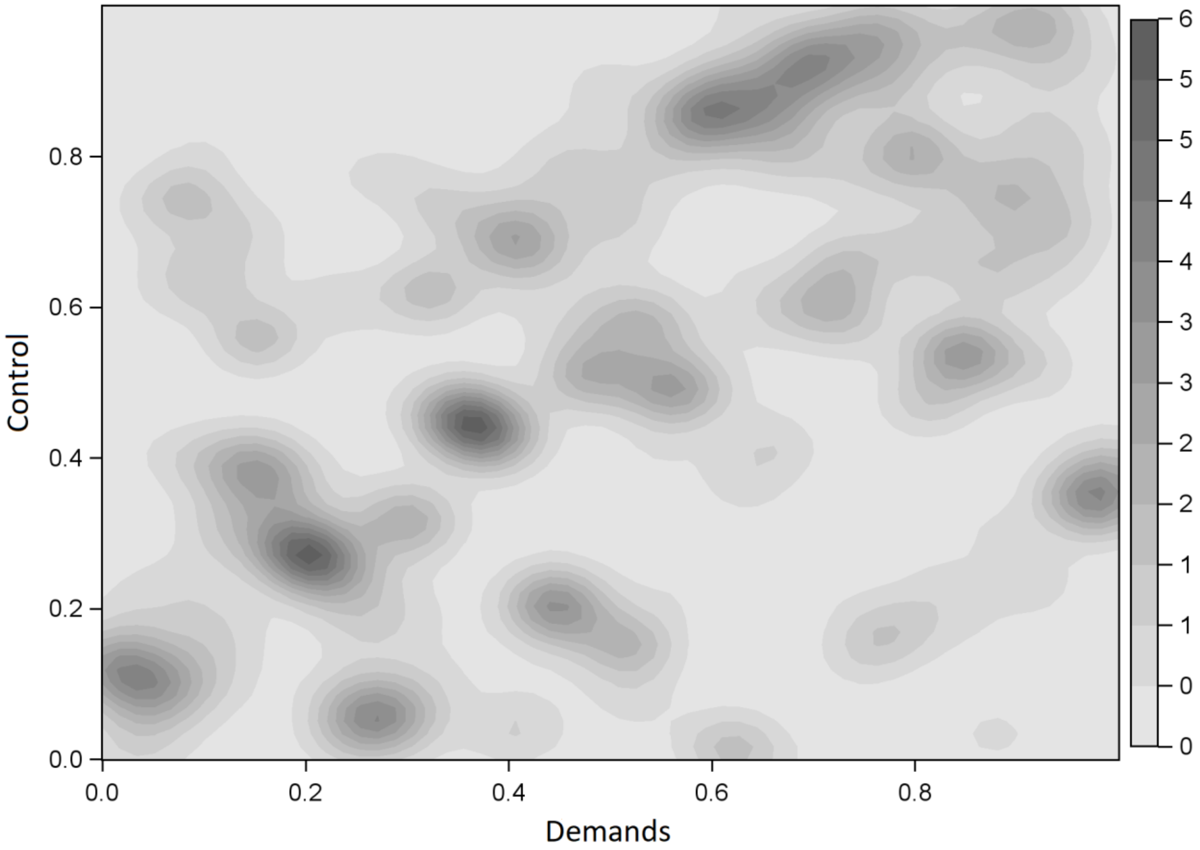

Farrants K, Head J, Framke E, Rugulies R, Alexanderson K. *Associations between combinations of job demands and job control among 616,818 people aged 55-64 in paid work with their labour market status 11 years later: a prospective cohort study.* Supplementary material.

b) 60-64

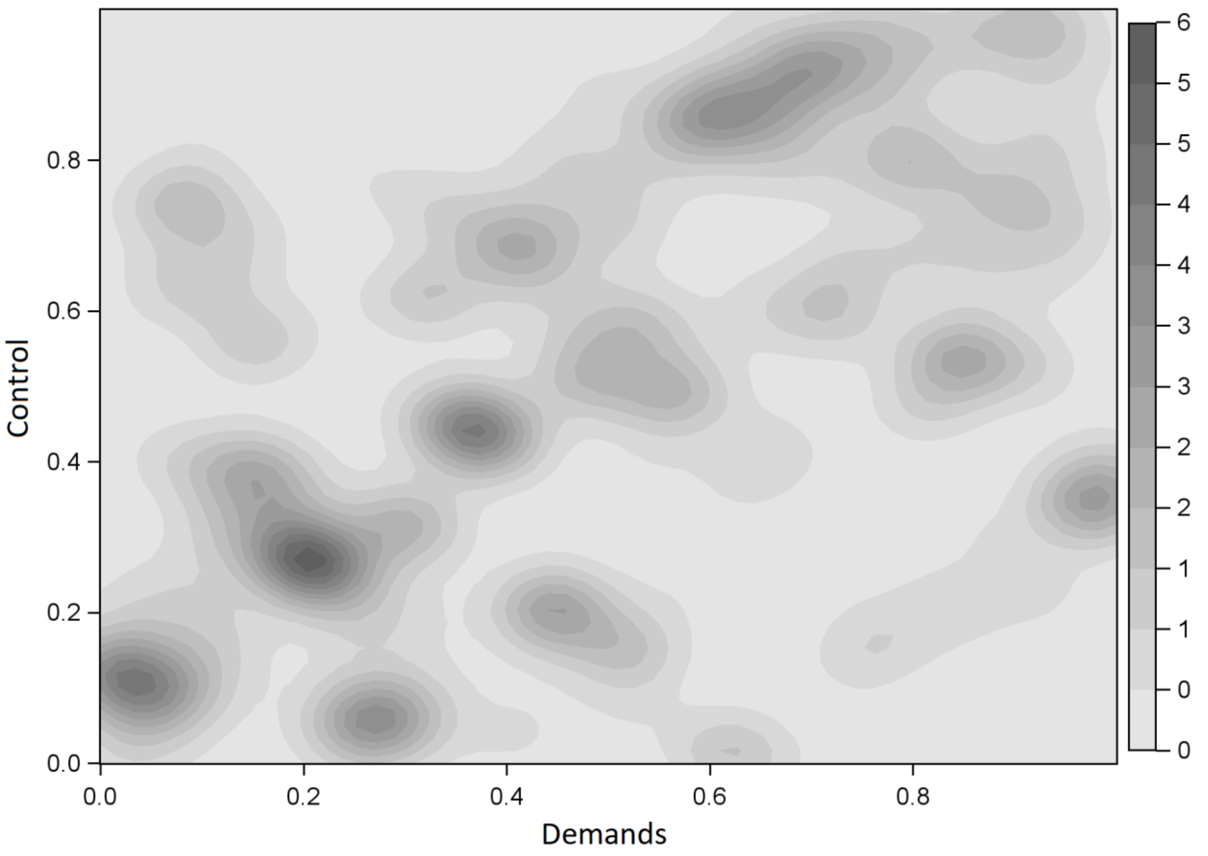

Farrants K, Head J, Framke E, Rugulies R, Alexanderson K. *Associations between combinations of job demands and job control among 616,818 people aged 55-64 in paid work with their labour market status 11 years later: a prospective cohort study.* Supplementary material.

**<u>Supplementary Figure 2.</u> Labour market status in 2012 by job demands and job control in 2001 among all in paid work in Sweden in 2001 by age group in 2001.**

**a). 55-59**

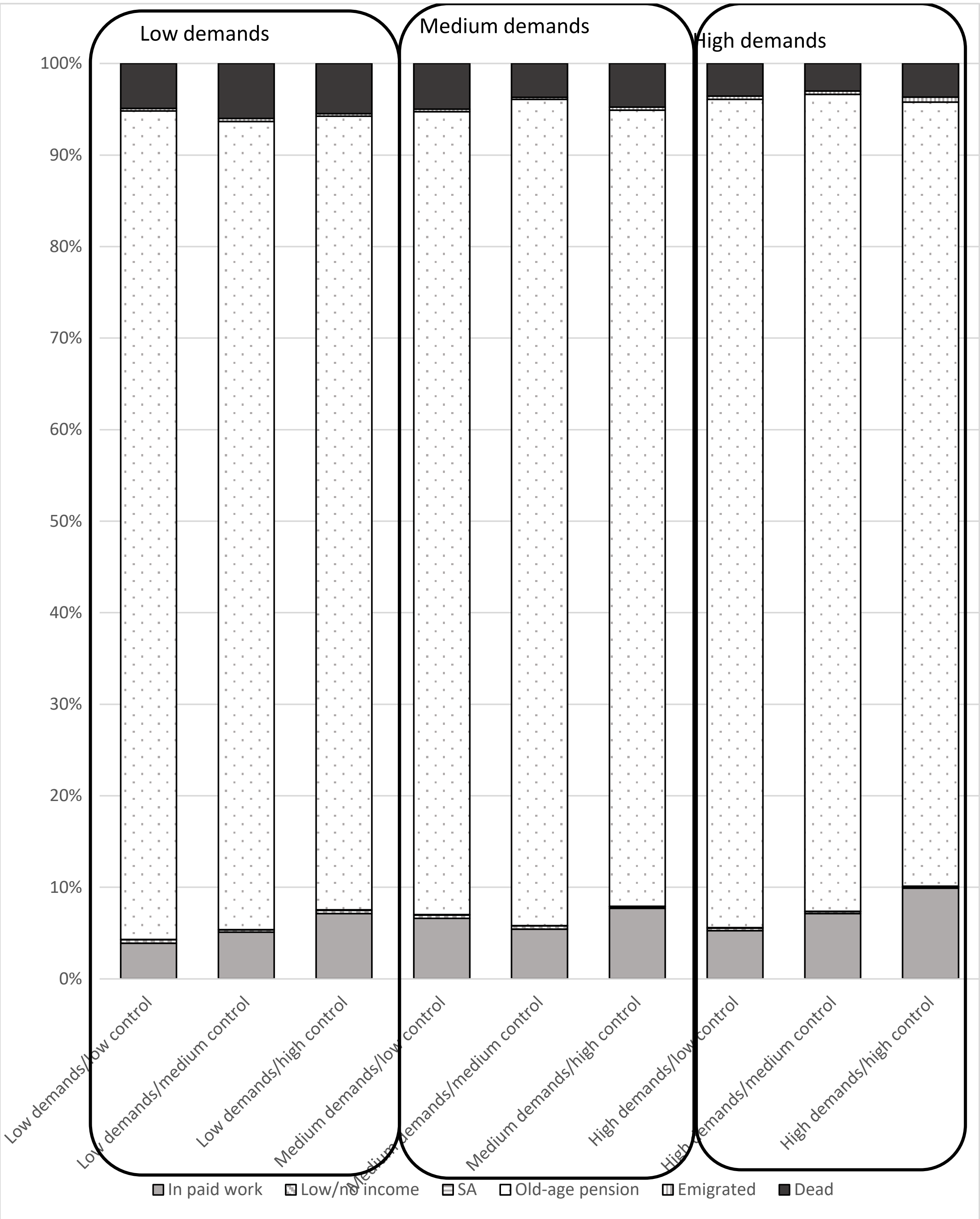

Farrants K, Head J, Framke E, Rugulies R, Alexanderson K. *Associations between combinations of job demands and job control among 616,818 people aged 55-64 in paid work with their labour market status 11 years later: a prospective cohort study.* Supplementary material.

**b.) 60-64**

Farrants K, Head J, Framke E, Rugulies R, Alexanderson K. *Associations between combinations of job demands and job control among 616,818 people aged 55-64 in paid work with their labour market status 11 years later: a prospective cohort study.* Supplementary material.

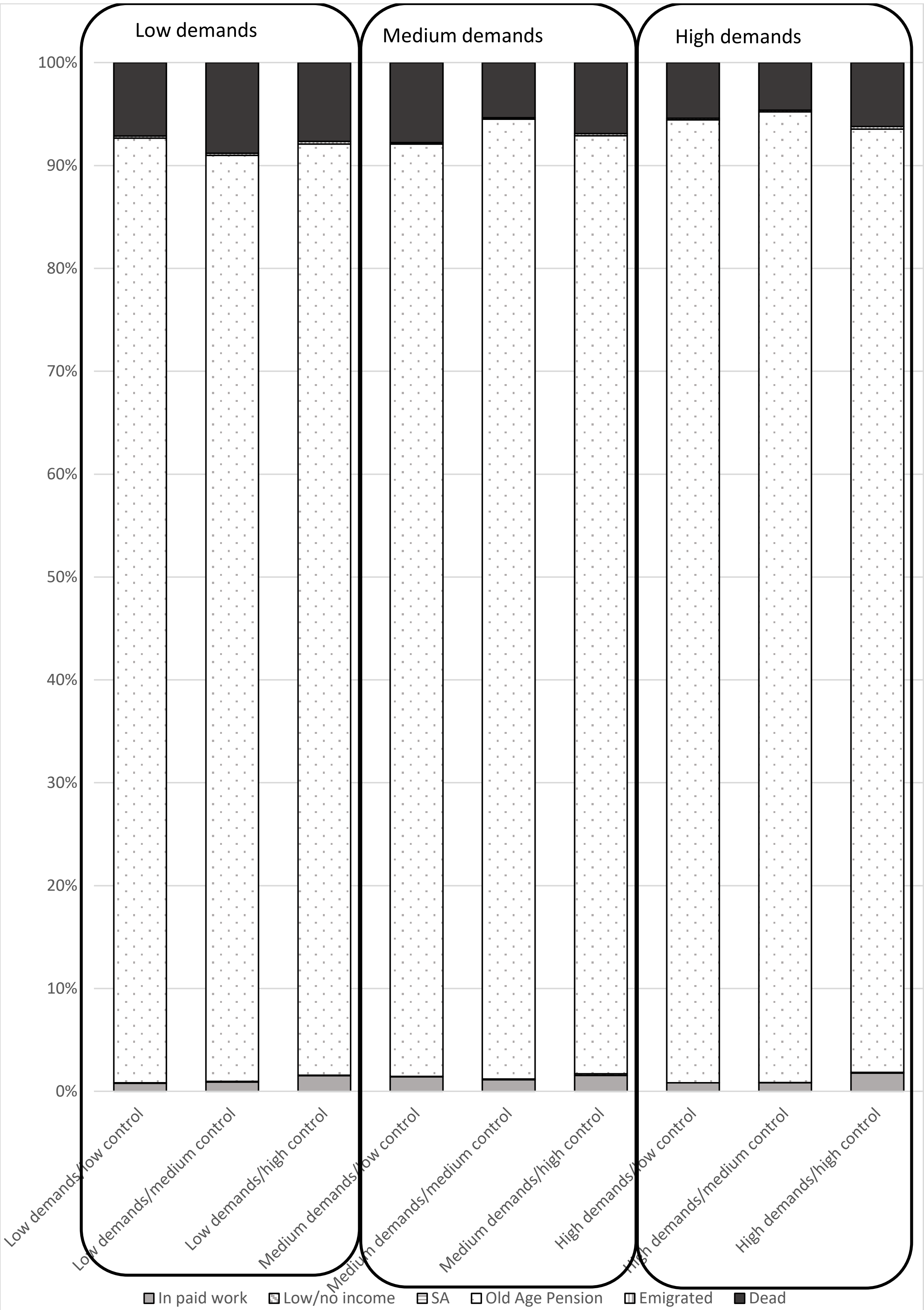

Farrants K, Head J, Framke E, Rugulies R, Alexanderson K. *Associations between combinations of job demands and job control among 616,818 people aged 55-64 in paid work with their labour market status 11 years later: a prospective cohort study.* Supplementary material.

**<u>Supplementary Figure 3. Labour market status in 2012 by age in 2012 among all in paid work in Sweden in 2001.</u>**

a) All

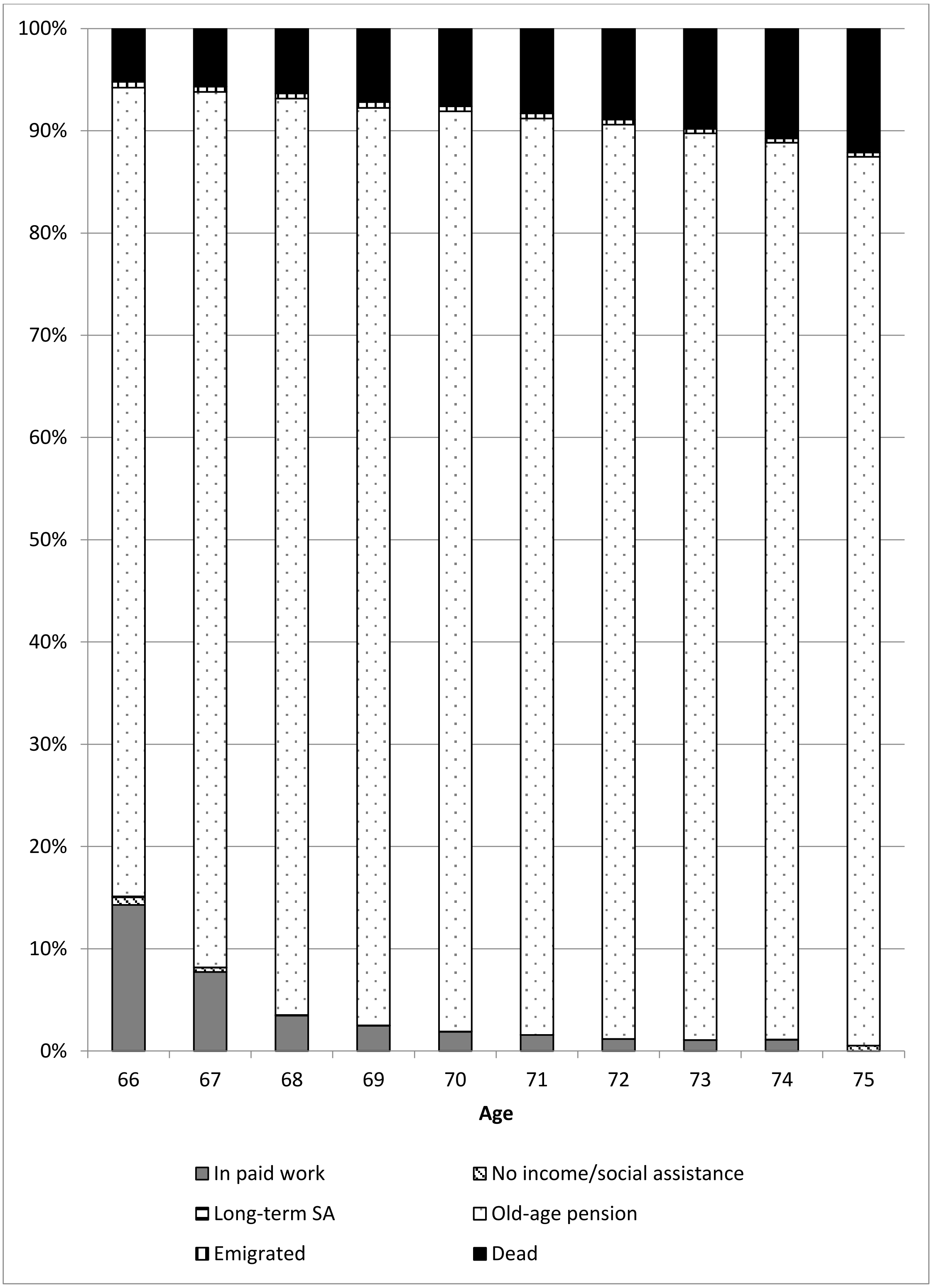

Farrants K, Head J, Framke E, Rugulies R, Alexanderson K. *Associations between combinations of job demands and job control among 616,818 people aged 55-64 in paid work with their labour market status 11 years later: a prospective cohort study.* Supplementary material.

b) women

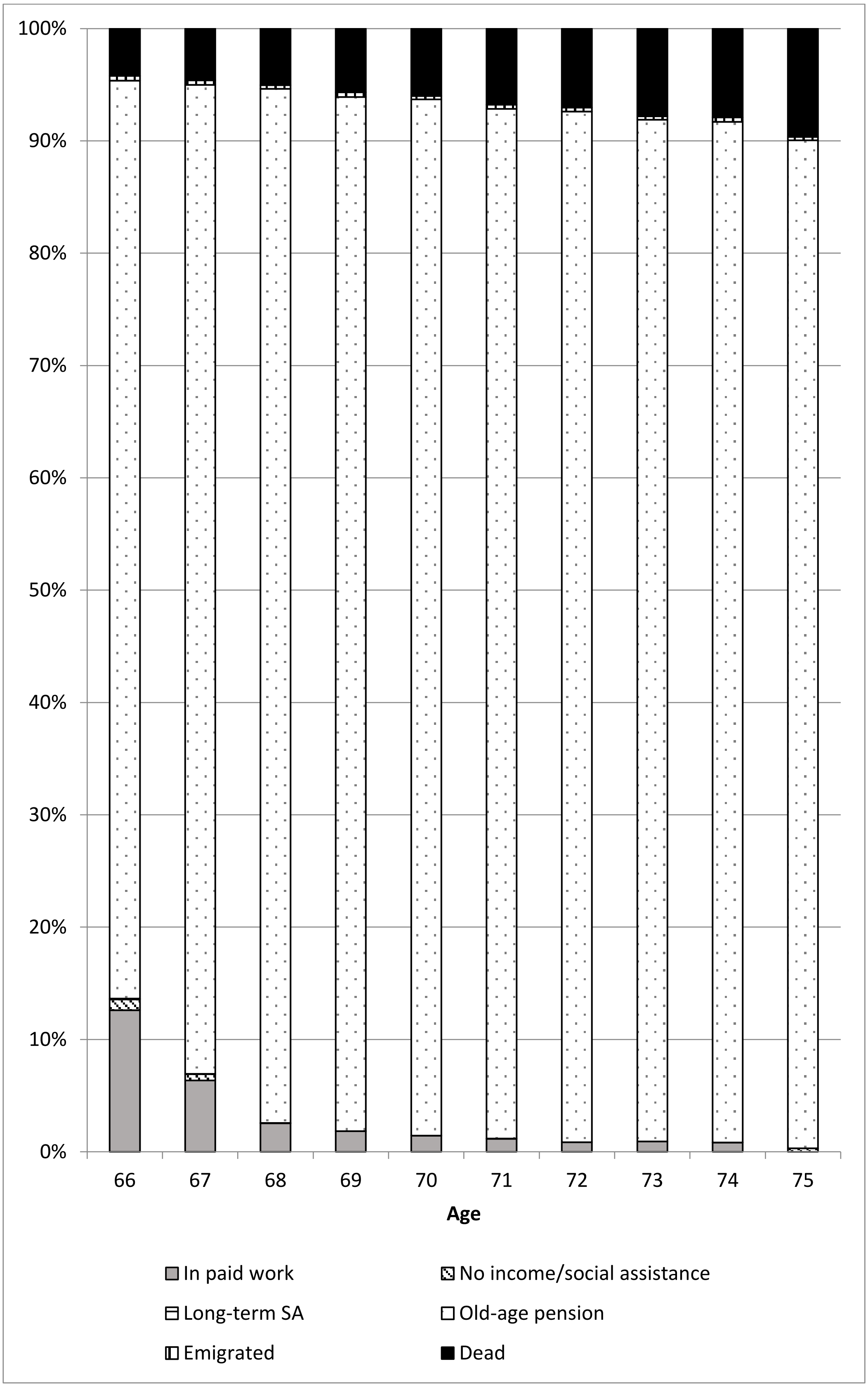
100%
90%
80%
70%
60%
50%
40%
30%
20%
10%
0%
66
67
68
69
70
71
72
73
74
75
Age
In paid work
No income/social assistance
Long-term SA
Old-age pension
Emigrated
Dead

Farrants K, Head J, Framke E, Rugulies R, Alexanderson K. *Associations between combinations of job demands and job control among 616,818 people aged 55-64 in paid work with their labour market status 11 years later: a prospective cohort study.* Supplementary material.

c) men

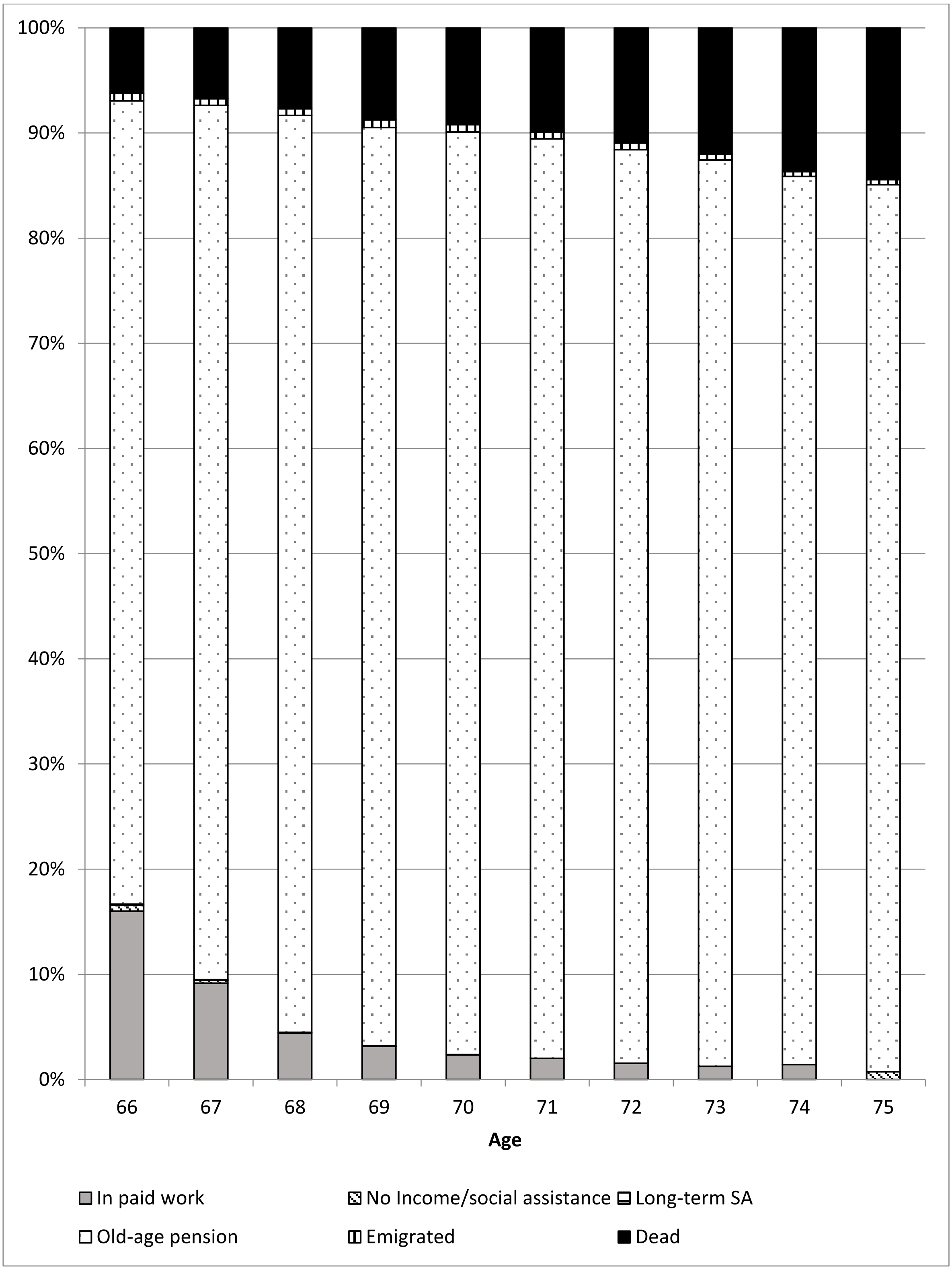

Farrants K, Head J, Framke E, Rugulies R, Alexanderson K. *Associations between combinations of job demands and job control among 616,818 people aged 55-64 in paid work with their labour market status 11 years later: a prospective cohort study.* Supplementary material.

**Supplementary table 1. Questions used to measure demands and control in the Job Exposure Matrix**

| |
|---|
| Demands: |
| • Is it at times so stressful that you don't have time to talk or even think about anything other than work? |
| • Do you sometimes have so much to do that you have to skip lunches, work overtime, or bring your work home? |
| • Does your work demand all your attention and concentration? |
| • To what degree of your working time do you have such difficult tasks that you need help? |
| Control: |
| • Are you partly able to determine yourself when different tasks are to be done (e.g. by choosing to work a little faster some days and a bit easier other days?) |
| • Do you have the possibility to determine your own pace at work? |
| • Are you able to take short breaks essentially any time to talk? |
| • Are you ever part of deciding your working arrangements (e.g. what shall be done, how it shall be done, or who shall work with you)? |
| • Does your work sometimes require you to repeat the same work task several times an hour? |

Farrants K, Head J, Framke E, Rugulies R, Alexanderson K. *Associations between combinations of job demands and job control among 616,818 people aged 55-64 in paid work with their labour market status 11 years later: a prospective cohort study.* Supplementary material.

**Supplementary table 2: Odds ratios (OR) and 95 % confidence intervals (CI) of labour market status (reference group=old age pension, total=537 882, women=280 631, men=257 251) in 2012 by combinations of job demands/control (reference group=medium demands/medium control) in 2001 among 616 818 individuals (313 100 women and 303 718 men) aged 55-59 and 60-64 in 2001 with income from work***

| | *In paid work* | | *No income/social assistance* | | *Sickness absence/disability pension* | | *Emigrated* | | *Dead* | |
|---|---|---|---|---|---|---|---|---|---|---|
| | 55-59 | 60-64 | 55-59 | 60-64 | 55-59 | 60-64 | 55-59 | 60-64 | 55-59 | 60-64 |
| Low demands/ low control | 0.75 (0.71-0.79) | 0.69 (0.58-0.81) | 1.10 (0.91-1.32) | 0.54 (0.28-1.06) | 0.70 (0.37-1.32) | None | 0.98 (0.78-1.22) | 1.27 (0.84-1.93) | 1.13 (1.07-1.20) | 1.20 (1.12-1.28) |
| Low demands/ medium control | 0.81 (0.76-0.85) | 0.61 (0.49-0.76) | 0.99 (0.74-1.31) | 0.49 (0.20-1.19) | 1.00 (0.49-2.01) | None | 0.84 (0.64-1.10) | 1.08 (0.64-1.83) | 1.04 (0.97-1.12) | 1.06 (0.97-1.16) |
| Low demands/ high control | 1.11 (1.03-1.19) | 1.09 (0.87-1.37) | 1.22 (0.90-1.65) | 0.38 (0.11-1.35) | 1.12 (0.48-2.66) | None | 0.72 (0.50-1.03) | 1.26 (0.70-2.27) | 1.11 (1.02-1.21) | 1.04 (0.94-1.16) |
| Medium demands/ low control | 1.16 (1.10-1.24) | 1.09 (0.91-1.32) | 1.26 (1.00-1.59) | 0.52 (0.21-1.28) | 1.49 (0.78-2.85) | None | 0.90 (0.68-1.19) | 0.78 (0.44-1.40) | 1.05 (0.98-1.13) | 1.15 (1.05-1.25) |
| Medium demands/medium control | 1 | 1 | 1 | 1 | 1 | None | 1 | 1 | 1 | 1 |
| Medium demands/ high control | 1.21 (1.06-1.18) | 1.05 (0.88-1.25) | 0.72 (0.55-0.94) | 1.43 (0.73-2.78) | 0.89 (0.45-1.76) | None | 1.00 (0.78-1.29) | 1.23 (0.76-2.00) | 0.99 (0.93-1.06) | 0.96 (0.89-1.05) |
| High demands/ low control | 0.82 (0.76-0.88) | 0.68 (0.51-0.89) | 0.77 (0.57-1.05) | None | 1.26 (0.56-2.78) | None | 1.16 (0.86-1.57) | 0.83 (0.42-1.63) | 0.95 (0.87-1.04) | 0.98 (0.87-1.01) |
| High demands/ medium control | 0.96 (0.90-1.01) | 0.67 (0.54-0.83) | 0.54 (0.41-0.70) | 0.31 (0.11-0.88) | 0.91 (0.46-1.81) | None | 1.18 (0.92-1.50) | 0.79 (0.47-1.31) | 0.90 (0.84-0.97) | 0.92 (0.83-1.01) |
| High demands/ high control | 1.28 (1.22-1.34) | 1.17 (0.99-1.37) | 0.58 (0.45-0.74) | 0.65 (0.32-1.35) | 0.88 (0.47-1.64) | None | 1.51 (1.22-1.88) | 1.72 (0.75-1.84) | 0.88 (0.83-0.94) | 0.97 (0.90-1.05) |

*Adjusted for age group, birth country, type of living area, family situation, educational level, labour market status in 2001

Farrants K, Head J, Framke E, Rugulies R, Alexanderson K. *Associations between combinations of job demands and job control among 616,818 people aged 55-64 in paid work with their labour market status 11 years later: a prospective cohort study.* Supplementary material.

**Supplementary table 3. Odds ratios (OR) and 95 % confidence intervals (CI) of labour market status (reference group=old-age pension, total=537 882, women=280 631, men=257 251) in 2012 by job demands (reference group=medium demands) and control (reference group=medium control) in 2001, among 616 818 individuals (313 100 women and 303 718 men) aged 55-64 in 2001 with an income from work, crude and adjusted***

| | *In paid work (n=29 210)* | | *No income/social assistance (n=1258)* | | *Sickness absence/disability pension (n=162)* | | *Emigrated (n=3133)* | | *Dead (n=45 173)* | |
|---|---|---|---|---|---|---|---|---|---|---|
| | Crude OR (95% CI) | Adjusted OR (95% CI) | Crude OR (95% CI) | Adjusted OR (95% CI) | Crude OR (95% CI) | Adjusted OR (95% CI) | Crude OR (95% CI) | Adjusted OR (95% CI) | Crude OR (95% CI) | Adjusted OR (95% CI) |
| *Total* | | | | | | | | | | |
| Low demands | 0.68 (0.66-0.70) | 0.79 (0.76-0.82) | 1.03 (0.91-1.17) | 0.96 (0.83-1.10) | 0.73 (0.49-1.08) | 0.74 (0.49-1.13) | 1.07 (0.97-1.17) | 1.01 (0.88-1.15) | 1.19 (1.17-1.22) | 1.07 (1.04-1.10) |
| Medium demands | 1 | 1 | 1 | 1 | 1 | 1 | 1 | 1 | 1 | 1 |
| High demands | 1.29 (1.26-1.33) | 0.99 (0.96-1.02) | 0.64 (0.56-0.75) | 0.60 (0.51-0.70) | 0.98 (0.68-1.40) | 0.90 (0.61-1.34) | 1.63 (1.50-1.78) | 1.29 (1.14-1.46) | 0.81 (0.79-0.83) | 0.92 (0.89-0.95) |
| Low control | 0.79 (0.76-0.81) | 1.00 (0.97-1.04) | 1.20 (1.06-1.37) | 1.16 (1.01-1.35) | 0.92 (0.62-1.35) | 1.09 (0.72-1.64) | 0.99 (0.90-1.08) | 1.07 (0.94-1.23) | 1.19 (.116-1.21) | 1.09 (1.06-1.12) |
| Medium control | 1 | 1 | 1 | 1 | 1 | 1 | 1 | 1 | 1 | 1 |
| High control | 1.56 (1.51-1.60) | 1.26 (1.22-1.30) | 0.81 (0.70-0.93) | 1.01 (0.87-1.18) | 1.17 (0.81-1.69) | 0.88 )0.59-1.32) | 1.55 (1.42-1.68) | 1.20 (1.06-1.36) | 1.10 (1.08-1.13) | 1.01 (0.98-1.04) |
| *Women* | *In paid work (n=12 249)* | | *No income/social assistance (n=762)* | | *Sickness absence/disability pension (n=64)* | | *Emigrated (n=1173)* | | *Dead (n=18 221)* | |
| Low demands | 0.72 (0.69-0.76) | 0.93 (0.88-1.00) | 1.06 (0.90-1.25) | 1.10 (0.87-1.38) | 0.49 (0.25-0.98) | 0.48 (0.21-1.10) | 1.13 (0.98-1.32) | 1.10 (0.84-1.45) | 1.21 (1.17-1.25) | 1.14 (1.08-1.21) |
| Medium demands | 1 | 1 | 1 | 1 | 1 | 1 | 1 | 1 | 1 | 1 |
| High demands | 1.37 (1.31-1.43) | 0.93 (0.88-0.98) | 0.61 (0.51-0.74) | 0.54 (0.43-0.68) | 1.09 (0.64-1.88) | 0.69 (0.36-1.32) | 1.48 (1.28-1.70) | 1.21 (0.97-1.51) | 0.83 (0.80-0.83) | 0.94 (0.88-0.99) |
| Low control | 0.67 (0.64- | 0.89 (0.84- | 1.16 (0.99- | 0.99 (0.79- | 0.78 (0.45- | 1.57 (0.80- | 0.90 (0.79- | 0.87 (0.68- | 1.25 (1.21- | 1.02 (0.97- |

| | | | | | | | | | | |
|---|---|---|---|---|---|---|---|---|---|---|
| | 0.70) | 0.95) | 1.35) | 1.24) | 1.36) | 3.09) | 1.02) | 1.12) | 1.30) | 1.08) |
| Medium control | 1 | 1 | 1 | 1 | 1 | 1 | 1 | 1 | 1 | 1 |
| High control | 1.46 (1.39-1.53) | 1.34 (1.27-1.41) | 1.05 (0.84-1.30) | 1.17 (0.93-1.48) | 1.38 (0.72-2.63) | 1.35 (0.69-2.65) | 1.36 (1.16-1.59) | 1.28 (1.03-1.58) | 1.03 (0.99-1.08) | 1.06 (1.00-1.12) |
| *Men* | *In paid work (n=16 931)* | | *No income/social assistance (n=496)* | | *Sickness absence/disability pension (n=98)* | | *Emigrated (n=1960)* | | *Dead (n=26 952)* | |
| Low demands | 0.64 (0.61-0.67) | 0.75 (0.70-0.77) | 0.99 (0.81-0.22) | 0.83 (0.66-1.04) | 0.88 (0.54-1.42) | 0.81 (0.48-1.37) | 1.01 (0.90-1.14) | 1.02 (0.86-1.21) | 1.16 (1.13-1.20) | 1.05 (1.01-1.09) |
| Medium demands | 1 | 1 | 1 | 1 | 1 | 1 | 1 | 1 | 1 | 1 |
| High demands | 1.24 (1.20-1.29) | 1.03 (0.99-1.07= | 0.70 (0.56-0.88) | 0.68 (0.53-0.80) | 0.90 (0.55-1.45) | 0.95 (0.57-1.60) | 1.73 (1.55-1.93) | 1.37 (1.17-1.60) | 0.79 (0.77-0.82) | 0.91 (0.87-0.95) |
| Low control | 1.02 (0.97-1.08) | 1.04 (0.99-1.10) | 1.28 (1.01-1.61) | 1.25 (0.97-1.59) | 1.15 (0.67-198) | 1.07 (0.61-1.89) | 1.16 (1.01-1.32) | 1.20 (1.00-1.43) | 1.19 (1.15-1.23) | 1.12 (1.07-1.16) |
| Medium control | 1 | 1 | 1 | 1 | 1 | 1 | 1 | 1 | 1 | 1 |
| High control | 1.51 (1.45-1.57) | 1.20 (1.15-1.25) | 0.82 (0.66-1.01) | 0.86 (0.67-1.09) | 0.89 (0.55-1.44) | 0.81 (0.47-1.39) | 1.31 (1.17-1.46) | 1.17 (0.99-1.39) | 0.85 (0.83-0.88) | 0.99-0.95-1.04) |

*Mutually adjusted and adjusted for age group, birth country, type of living area, family situation, educational level, labour market status in 2001

Farrants K, Head J, Framke E, Rugulies R, Alexanderson K. *Associations between combinations of job demands and job control among 616,818 people aged 55-64 in paid work with their labour market status 11 years later: a prospective cohort study*. Supplementary material.
